# Comparative analysis of non-adherence to medication treatment for systemic arterial hypertension in urban and rural populations^1^

**DOI:** 10.1590/0104-1169.0144.2520

**Published:** 2015

**Authors:** Patricia Magnabosco, Eliana Cavalari Teraoka, Edward Meirelles de Oliveira, Elisangela Aparecida Felipe, Dayana Freitas, Leila Maria Marchi-Alves

**Affiliations:** 2 Doctoral student, Escola de Enfermagem de Ribeirão Preto, Universidade de São Paulo, WHO Collaborating Centre for Nursing Research Development, Ribeirão Preto, SP, Brazil. Assistante Professor, Universidade Federal de Uberlândia, Uberlândia, MG, Brazil; 3 Doctoral student, Escola de Enfermagem de Ribeirão Preto, Universidade de São Paulo, WHO Collaborating Centre for Nursing Research Development, Ribeirão Preto, SP, Brazil. Scholarship holder from Coordenação de Aperfeiçoamento de Pessoal de Nível Superior (CAPES), Brazil; 4 Doctoral student, Faculdade de Filosofia, Ciências e Letras de Ribeirão Preto, Universidade de São Paulo, Ribeirão Preto, SP, Brazil. Scholarship holder from Coordenação de Aperfeiçoamento de Pessoal de Nível Superior (CAPES), Brazil; 5 Social worker, Escola Eurípedes Barsanulfo, Sacramento, MG, Brazil; 6 Doctoral student, Escola de Enfermagem de Ribeirão Preto, Universidade de São Paulo, Centro Colaborador da OMS para o Desenvolvimento da Pesquisa em Enfermagem, Ribeirão Preto, SP, Brazil. RN, UBS/PSF Gilberto Arthur Abate, Prefeitura Municipal de Frutal, Frutal, MG, Brazil; 7 PhD, Professor, Escola de Enfermagem de Ribeirão Preto, Universidade de São Paulo, Centro Colaborador da OMS para o Desenvolvimento da Pesquisa em Enfermagem, Ribeirão Preto, SP, Brazil

**Keywords:** Hypertension, Medication Adherence, Urban Population, Rural Population

## Abstract

**OBJECTIVE:**

to evaluate the indexes and the main factors associated with non-adherence to medication treatment for systemic arterial hypertension between urban and rural areas.

**METHOD:**

analytical study based on an epidemiological survey with a sample of 247 hypertensive residents of rural and urban areas, with application of a socio-demographic and economic questionnaire, and treatment adherence assessment. The Pearson's Chi-square test was used and the odds ratio (OD) was calculated to analyze the factors related to non-adherence.

**RESULTS:**

the prevalence of non-adherence was 61.9% and it was higher in urban areas (63.4%). Factors significantly associated with non-adherence were: male gender (OR=1.95; 95% CI 1.08-3.50), age 20-59 years old (OR=2.51; 95% CI 1.44-4.39), low economic status (OR=1.95; 95% CI 1.09-3.47), alcohol consumption (OR=5.92, 95% CI 1.73-20.21), short time of hypertension diagnosis (OR=3.07; 95% CI 1.35-6.96) and not attending the health service for routine consultations (OR=2.45; 1.35-4.42).

**CONCLUSION:**

the socio-demographic/economic characteristics, lifestyle habits and how to relate to health services were the factors that presented association with non-adherence regardless of the place of residence.

## Introduction

Non-adherence to medication therapy is a worldwide problem by reducing the therapeutic results, especially chronic diseases, and increasing the costs of health systems^(^ 1 ^)^.

Conceptually, non-adherence should be assumed as a multi-dimensional construct. It is related not only to medication intake or not, but to how the patients "manage" their treatment: behavior in relation to dose, time, frequency and duration^(^ 2 ^)^.

Few studies in Brazil and around the world describe the adherence rates among hypertensive patients, especially in rural areas. Most investigations have been focused on the evaluation in urban centers and these data cannot be extended to rural areas, as populations have very different demographic characteristics, food and cultural habits, occupation types and access to health care^(^ 3 ^)^.

The literature shows that the prevalence of non-adherence of people with Systemic Arterial Hypertension (SAH) to medication treatment is quite varied^(^ 4 ^)^. Research conducted with hypertensive patients in outpatient clinics of Brazilian hospitals showed a percentage of non-adherence of 86.7% in São José do Rio Preto (SP)^(^ 5 ^)^ and 12.7% in Ribeirão Preto (SP)^(^ 6 ^)^. In the primary care of a small town in Rio Grande do Sul, the non-adherence rate was 34.3%^(^ 7 ^)^. In Teresina (PI), non-adherence was 26.8%^(^ 8 ^)^ and, in Maringá (PR), it was equal to 64.0%^(^ 9 ^)^.

Studies in rural areas of different countries have also shown varying rates of non-adherence to medication treatment for SAH. Rates of 66.0% in Turkey^(^ 10 ^)^ and 60.1% in the United States^(11) ^were found. In Brazil, a study conducted in the state of Minas Gerais showed non-adherence rate of 28%^(^ 12 ^)^.

The world report shows that this variability in the frequency of adherence depends on the method used for its estimation, population characteristics investigated and the sample size^(^ 1 ^)^.

Regarding the factors that influence treatment adherence, researchers point out multiple causes, i.e. adherence depends on the disease (chronicity, absence of symptoms and late consequences), treatment (medication consumed), characteristics and beliefs of people (gender, age, ethnicity, marital status, education and socio-economic status), life habits, cultural aspects (lack of perception of the seriousness of the disease, ignorance, illness in the family context and self-esteem) and how people with SAH relate with the health service^(^ 1 ^)^.

Thus, given the diversity of contexts between residents of urban and rural areas, this study aims to evaluate the indexes and the main factors associated with non-adherence to medication treatment for SAH between urban and rural areas of a Brazilian city.

## Method

The study was conducted in a city located in the Triângulo Mineiro, region of Alto Paranaíba in the State of Minas Gerais, which has a population of 23,880 inhabitants, with 19,278 living in the urban area and 4,602 in the rural area. The population aged 20 years or older is 14,217 people, with 12,974 in the urban and 1,243 in the rural area^(^ 13 ^)^.

The coverage of the Family Health Strategy (FHS) in the city is 85.9% and it has six teams, one with only rural activities, covering approximately 700 families distributed in six micro-areas. The long distances and difficult traffic conditions and mobility between areas are some of the main challenges for interaction with rural communities, historically relegated to low priority.

For this study, we investigate a population of 1,243 adults registered in the rural FHS and 4,690 people enrolled in two FHS teams in the urban area. The sample size determination was based on the population proportion estimate, using a SAH prevalence rate equal to 44%, maximum value according to studies conducted in Brazil^(^ 14 ^)^. Individuals were selected through random sampling that included 5,933 adult inhabitants resident in rural and urban areas, with correction for finite populations and adjustment for a refusal population of 20%, respecting the population density of each area. The confidence interval was set as 95% and the design error as 2.5%.

For each area covered by the FHS teams, a sample corresponding to 563 participants from urban and 153 from rural areas was estimated. For the data collection procedure, a sample draw was performed in four stages. The sampling units of the first stage were carried out according to the FHP coverage areas of urban and rural regions of the municipality.

In the urban area, the second stage comprised sampling by street, the third by residences, and the fourth by choosing a resident. To maintain and ensure the random characteristic of the sample, it was determined that the selection of individuals would include the individual with the first upcoming birthday, from the date of the interview, among the inhabitants of the residence drawn.

The selection of participants in the rural area occurred by random drawing from the numeric register of families in the FHS, registered in the Primary Care Information System (SIAB) of the Municipal Health Secretariat. For the selection of the individuals, the criterion valid for the urban population was used.

Preliminarily, the individuals were informed about the objectives and procedures of the study and, next, they were invited to participate in the study. The data collection was carried out after signing the Informed Consent Form (ICF). The sample included individuals aged 20 years or older who reported having SAH and excluding pregnant women and people with serious psychiatric illness or mental disability informed by a relative. These criteria determined the subject studied in 247 hypertensive patients (194 in urban areas and 53 in rural areas).

Data collection was carried out between January and August 2013, through a semi-structured questionnaire and instruments that permitted the evaluation of socio-demographic and economic variables, risk factors for SAH, knowledge of hypertensive condition, medications in use, access to health services and reasons why participants seek care.

Non-adherence to medication treatment of SAH was the dependent variable addressed and the independent variables of interest were: gender; age (young/adult or elderly); skin color reported according to the perception of the individual (white or non-white); years of education (< 8 years or ≥ 8 years); economic status (classified based on the criteria of the Economic Classification of Brazil according to the Associação Brasileira de Empresas de Pesquisa^(^ 15 ^)^, which takes education and consumer goods for reference, where the variation occurs between Class A, which is the best stratum, up to class E, with the most unfavorable conditions); sedentary lifestyle (not performing physical activity at least three times/week for at least 30 minutes/day); smoking (consumption of at least one cigarette/day); alcohol consumption (consumption of more than 30 g of ethanol/day for men and 15g/day for women)^(^ 14 ^)^; time of diagnosis of SAH (up to 3 years or ≥ 3 years), according to criteria adopted by the authors of the non-adherence measurement instrument used in this study^(^ 16 ^)^; difficulty to access the health services; type of health service used (health insurance/private or SUS); reason why the health service was searched (only in emergency cases, for routine consultations - at least one medical visit every six months^(^ 14 ^)^ - or to get medications) and amount of anti-hypertensive pills prescribed a day.

For the assessment of non-adherence to treatment of SAH, the instrument "Questionnaire of Adherence to Medications - Qualiaids" (QAM-Q) was used, developed and validated in Brazil^(^ 2 ^)^. The questionnaire was developed to address the act (if the individual takes and how much medication is taken), the process (such as how medication is taken in seven days, if he/she skips, if he/she takes it abnormally, if he/she has "breaks") and the adherence results (in this case, if his/her pressure was under control).

To sort the interviewee as non-adherent a composite measure was constructed, in which the presence of one of these conditions was sufficient: either not taking the correct amount (80% - 120% of the prescribed doses), or not taking it the right way (without "breaks", "erratic intake", abandonment or "partial adherence"), or to report that his/her blood pressure was altered^(^ 2 ^)^.

To analyze the association between the dependent variable (non-adherence to treatment of SAH) and socio-demographic and economic variables, clinical/treatment/lifestyle and access to health services, Pearson's Chi-square test was used. The odds ratio (OD) was calculated with the respective 95%confidence intervals for each variable studied. The significance level was set as α=0.05. The software SPSS Windows *Statistical Package for the Social Sciences* (SPSS), version 17.0 was used.

This study was approved by the Research Ethics Committee of the University of São Paulo at Ribeirão Preto College of Nursing (EERP-USP) under protocol 188/2012.

## Results

The prevalence of non-adherence to medication treatment of SAH as a combined measure of QAM-Q was 153 (61.9%), being higher in the urban area, 123 (63.4%), than in rural area, 30 (56.6%). No association was found between place of residence and non-adherence to treatment (OD= 1.32; 95% CI 0.71-2.46).

The distribution frequencies of socio-demographic and economic characteristics, clinical/treatment/lifestyle and access to health services in urban and rural populations are shown in Tables 1 and 2.

**Table 1 -:** Distribution of hypertensive patients according to socio-demographic/economic and lifestyle variables of urban and rural population. Sacramento, MG, Brazil, 2013

Study variable %		Rural (n=53) %	Urban (n=194) %	Total Population (n=247)
Gender				
	Female	56.6	72.7	69.2
	Male	43.4	27.3	30.8
Age Group (years)				
	≥ 60 years old	39.6	66.0	60.3
	Up to 59 years old	60.4	34.0	39.7
Skin color				
	White	92.5	73.2	77.3
	Non-white	7.5	26.8	22.7
Education				
	< 8 years of study	84.9	71.6	74.5
	≥ 8 years of study	15.1	28.4	25.5
Economic Status*				
	A/B	13.2	29.4	25.9
	C/D/E	86.8	70.6	74.1
Smoking^†^				
	No	84.9	85.6	85.4
	Yes	15.1	14.4	14.6
Alcohol consumption^‡^				
	No	88.7	90.2	88.7
	Yes	11.3	9.8	11.3

* Economic Status A/B (more favorable) and C/D/E (less favorable)

† Smoking (consuming at least one cigarette/day)

‡ Alcohol consumption (consumption of more than 30 g ethanol/day for men and 15g/day for women).

**Table 2 -:** Distribution of hypertensive patients according to clinical variables/treatment and access to health services in urban and rural population. Sacramento, MG, Brazil, 2013

Study variable %		Rural (n=53) %	Urban (n=194) %	Total (n=247)
Time of diagnosis (Systemic Arterial Hypertension)				
	More than 3 years	77.4	77.8	83.0
	Up to 3 years	22.6	22.2	17.0
Pills/day				
	1 to 2	48.1	53.8	52.3
	≥ 3	51.9	46.2	47.7
Service Type				
	Health Insurance/Private	3.8	22.7	23.9
	Brazilian Unified Health System	96.2	77.3	76.1
Difficulty to access				
	No	60.4	68.8	66.4
	Yes	39.6	32.0	33.6
Reason for seeking the health service				
	Routine visit*	62.3	64.4	63.6
	To get medication	5.7	7.6	3.6
	Only emergency	32.1	28.8	32.8

* Routine visit (at least one medical visit every 6 months)

Non-adherence measured by the proportion of dosages of QAM-Q was 53 (27.3%) in the urban population and 16 (30.2%) in rural areas. As regards taking the medication measured by the QAM-Q, 82 (42.3%) people were considered non-adherent in the urban area and 21 (39.6%) in rural areas; of these, 12 (6.2%) in the urban area and three (5.7%) in rural areas reported taking medications at changed times; 10 (5.2%) people in the urban area had "breaks" in the use of antihypertensive medications, but none among hypertensive patients in rural areas; 20 (10.3%) took it erroneously in urban areas and six (11.3%) in rural areas; 15 (7.7%) had half adherence in urban areas and seven (13.2%) in rural areas; six (3.1%) exchanged dose levels in the urban and one (1.9%) in the rural area; three (1.5%) partially abandoned medication treatment in the urban and none in the rural area; 21 (10.8%) totally abandoned the medication in the urban and five (9.4%), in the rural population. Regarding treatment results, 87 (44.8%) residents in the urban and 20 (37.7%) in the rural area reported that their blood pressure was altered the last time it was measured.

The frequency distribution and association between non-adherence and the variables studied are presented in Tables 3 and 4.

**Table 3 -:** Univariate analysis of the association between non-adherence and socio-demographic/economic and lifestyle characteristics. Sacramento, MG, Brazil, 2013

Study variable		Non-adherence(%)				Likelihood Ratio	Confidence Interval (95%)	p*
		Rural (n=53)	Urban (n=194)	Total (n=247)				
Gender							(1.08-3.50)	0.025‡
	Female	50.0	58.9	57.3		1		
	Male	65.2	75.5	72.4		1.95		
Age Group (years)							(1.44- 4.39)	0.001‡
	≥ 60 years old	47.6	54.7	53.7		1		
	Up to 59 years old	62.5	80.3	74.5		2.51		
Skin color							(0.74-2.62)	0.301
	White	59.2	60.6	60.2		1		
	Non-white	25.0	71.2	67.9		1.39		
Education							(0.66-2.17)	0.553
	< 8 years of study	55.6	62.6	60.9		1		
	≥ 8 years of study	62.5	65.5	65.1		1.19		
Economic status ^†^							(1.09-3.47)	0.023‡
	A/B	42.9	50.9	50.0		1		
	C/D/E	58.7	68.6	66.1		1.95		
Smoking^‡^							(0.90-4.51)	0.081
	No	55.9	60.8	59.7		1		
	Yes	62.5	78.6	75.0		2.02		
Alcohol consumption^§^							(1.73-20.21)	0.002‡
	No	53.2	60.6	58.4		1		
	Yes	83.3	89.5	89.3		5.92		

* Pearson's chi-square test (p <0.05)

† Economic Status A/B (more favorable) and C/D/E (less favorable)

‡ Smoking (consuming at least one cigarette/day)

§ Alcohol consumption (consumption of more than 30 g ethanol/day for men and 15g/day for women)

**Table 4 -:** Univariate analysis of the association of non-adherence and clinical characteristics/treatment and access to health services. Sacramento, MG, Brazil, 2013

Study variable		Non-adherence(%)				Likelihood Ratio	Confidence Interval (95%)	p*
		Rural (n=53)	Urban (n=194)	Total (n=247)				
Time of diagnosis							(1,35- 6,96)	0,005
	More than 3 years	53.7	58.3	58.0		1		
	Up to 3 years	66.7	81.4	81.0		3.07		
Pills/day							(0.67-1.91)	0.632
	1 to 2	40.0	63.6	58.5		1		
	More than 3	70.4	58.8	61.6		1.13		
Service Type							(0.92-3.01)	0.089
	Health insurance/Particular	50.0	50.0	52.5		1		
	Brazilian Unified Health System	56.9	67.3	64.9		1.67		
Difficulty to access							(0.41-1.22)	0.222
	No	53.1	67.4	64.6		1		
	Yes	61.9	54.8	56.6		0.71		
Reason for seeking the health service							(0.44-0.84)(1.35-4.42)	0.003
	Routine visit^†^	48.5	56.0	54.1		1		
	To get medication	100.0	59.3	77.8		2.20		
	Only emergency	64.7	78.1	75.3		2.45		

* Pearson's chi-square test (p <0.05)

† Routine visit (at least one medical visit every six months)

The reasons reported by the hypertensive patients relative to non-adherence to medication treatment were: absence of symptoms (51.3%), side effects (21.8%), forgetfulness (16.8%), economic factors (5.9%) and others (4.2%).

The factors that hampered the access to health services were: distance in relation to the local of care (77.6%) and lack of vacancies (15.6%) according to the residents of the rural area, and availability of vacancies (46.0%) and limited mobility (44.4%) according to urban population.

## Discussion

The rate of non-adherence to SAH treatment presented in this work is within the percentage limits reported in other studies conducted in Brazil^(^ 2 ^,^ 5 ^,^ 7 ^-^ 9 ^)^.

Among the three non-adherence measurement parameters used by the QAM-Q instrument, our findings indicate that the process of taking medication was the main responsible for non-adherence to treatment among hypertensive patients in the rural area; in the urban population, it was highlighted the "treatment result". The use of "treatment result" as a non-adherence to treatment variable may represent a limitation or bias of the study, since the reporting of increased blood pressure does not necessarily mean that the participant did not adhere to medication treatment. The change in blood pressure levels may be related to inappropriate clinical management (unsatisfactory dosage, ineffective drug) or incorrect or insufficient blood pressure measurement (single measurement on a single day).

In the rural area, there was a prevalence of younger people who use more than three pills a day. However, the percentages related to socio-demographic characteristics, economic, clinical, related to lifestyle and access to health care may be more significant for both populations (Tables 1 and 2).

Both in rural and in urban areas, more than half of the hypertensive patients did not adhere to the medication treatment of SAH. A difference of about seven percentage points between adherence rates was observed though, especially in urban areas. In line with our results, a study conducted in the State of Minas Gerais (Brazil) found a rate of non-adherence to medication treatment of SAH six percent higher in the urban area compared to the rural area^(^ 12 ^)^. Some researchers believe that the fact can be attributed to the influence of socio-economic and cultural aspects^(^ 17 ^)^.

All six variables that are statistically associated with non-adherence (male, age up to 59 years old, low economic status, alcohol consumption, shorter time of SAH and reason for seeking the health service - only in emergencies) showed higher prevalence among hypertensive patients living in the urban area (Tables 3 and 4).

Our findings indicated that hypertensive females adhered more to treatment in both urban and rural areas. The males showed a risk of non-adherence 1.95 higher when compared to women, with a statistically significant difference (Table 3). Studies have pointed out that women perceive and report their health problems more obstinately than men, and that they seek health services more often and therefore follow the prescription better ^(^ 16 ^)^.

In terms of age, several studies have also shown poor adherence among non-elderly^(^ 9 ^,^ 14 ^,^ 18 ^)^, both in rural and urban areas, where people 20-59 years old had 2.51 times higher risk of not adhering to drug treatment than participants of 60 years old or older. It has been argued that the characteristic of asymptomatic SAH can cause certain indifference in younger people regarding the control of the disease, increasing the risks of severe complications and mortality by cardiovascular disease^(^ 9 ^)^.

We also found that individuals with lower economic status, belonging to economic status C, D or E, showed a risk of 1.95 higher of non-adherence to treatment, compared to those in status A or B. This event has already been reported by other researchers^(^ 2 ^,^ 18 ^)^, according to whom the socio-economic situation strongly influences the adherence, and are related not only to the purchasing power of medication, but also to educational, cultural and social aspects^(^ 2 ^)^.

Although some investigations have found that, the higher the education degree, the higher will be the level of adherence to medication treatment^(^ 10 ^,^ 19 ^)^, in this study we did not observe this relationship.

Alcohol consumption appears to be associated with non-adherence to medication treatment of SAH, whether in rural or urban area. According to our findings, those who consume alcohol had a risk of non-adherence almost six times higher than the hypertensive people who did not drink alcohol. The fear of possible undesirable effects caused by the association of antihypertensive medication with alcoholic beverages was a major report presented in a cross-sectional study with 401 patients, conducted in different centers of Bahia State in order to analyze the reasons that lead patients to non-adherence to treatment of SAH^(^ 20 ^)^.

Differently than stated in the literature, which argues that a high number of medicines used tends to decrease adherence^(^ 16 ^,^ 21 ^)^, our results did not show association between polypharmacy and adherence to medication treatment of SAH.

These research data indicate that hypertensive individuals with less time of diagnosis of SAH (up to three years) had higher non-adherence rates than those with longer time (over three years) (Table 4). The results of another study with hypertensive people reiterate that, the shorter the duration of the disease and pharmacological treatment, the higher the non-adherence rate^(^ 16 ^)^.

According to the hypertensive patients who composed our sample, the "absence of symptoms" was the main reason for non-adherence to medication treatment, both in rural and urban areas. For many researchers^(^ 10 ^,^ 16 ^)^, asymptomatic characteristic of the disease and the need for lifelong treatment are remarkable events that contribute to non-adherence to treatment. A study^(^ 22 ^)^ conducted with 353 patients at a University Hospital in the state of São Paulo pointed out that the asymptomatic nature of SAH contributed to 36% of non-adherence.

We also found that the majority of the population, both in urban and rural areas, reported using the Brazilian Unified Health System (SUS). The difficulty of access to health care was mentioned by participants in both areas, but prevailed among the residents of rural areas, for whom the distance to be covered is the element that contributes imperatively to this restriction. Those attending the service for routine consultations adhered more to treatment than those who visited the service only to get medications or in case of emergencies, both in rural as in urban areas (Table 4).

The attendance to appointments is crucial for the control of hypertension, because it brings individual motivation and this, in turn, leads to certain attitudes that favor the reduction of blood pressure, and adherence to medication treatment^(^ 5 ^)^. A study of 245 hypertensive patients in the outpatient clinic of a university hospital showed that, among the 220 regular hypertensive patients, i.e. patients who attended the consultations within 30 days of the previously scheduled date for their return, 200 (90.9%) said they were taking the medication according to prescription^(^ 6 ^)^.

We also point out that, despite the great geographical distance between rural areas and health services reported by participants in our study, no association was found between the difficulty of access and non-adherence, which can be explained by the service provided by rural FHS. This team is present once a week in each village and, in all of them, there is a health center with daily presence of the community agent and nursing assistant, enabling, even incipiently, contact with the health service. Even with a number of difficulties in the political-administrative and financial context, the great expansion of primary health care in Brazil through the FHS has caused a positive impact on the health status and general health of the population^(^ 23 ^)^.

According to the World Health Organization^(^ 1 ^)^, however, age, gender, education, occupation, marital status, religion, ethnicity and urban versus rural life have not been associated with permanent adherence. Some authors^(^ 16 ^)^ reported that non-adherence to treatment of SAH is more closely related to non-recognition of disease problems, to being asymptomatic than the difficulty of access to health services, a fact also observed in this study.

## Conclusion

Our findings revealed high rates of non-adherence to medication treatment of SAH in urban and rural areas, with the more significant rates in the urban population. Males aged between 20 and 59 years, low economic status, alcohol consumption, short time of diagnosis and no seek for health services for routine consultations were the factors that were associated with non-adherence to medication treatment for SAH. The fact of living in urban or rural areas did not influence the adherence to SAH treatment of. The factors related to the characteristics and beliefs, life habits and how hypertensive patients relate to health services, presented association with non-adherence. Knowledge of the main factors related to non-adherence to medication treatment of SAH and the identification of vulnerable community groups is of great value to guide the planning of health policies, enabling the prevention of complications and preventing the impairment of human health.

## References

[1] World Health Organization (2003). Adherence to long-term therapies: evidence for action.

[2] Santa Helena ET, Nemes MIB, Eluf-Neto J (2008). Desenvolvimento e validação de questionário
multidimensional para medir não-adesão ao tratamento com
medicamentos. Rev Saúde Pública..

[3] Balieiro HM, Osugue RK, Rangel SP, Brandão R, Balieiro TL, Bernardez S (2009). Perfil clínico-demográfico e indicadores de qualidade da
insuficiência cardíaca em uma área rural. Arq Bras Cardiol..

[4] Naderi SH, Bestwick JP, Wald DS (2012). Adherence to drugs that prevent cardiovascular disease:
meta-analysis on 376,162 patients. Am J Med..

[5] Dosse C, Cesarino CB, Vilela Martin JF, Castedo, MCA (2009). Factors associated to patients' noncompliance with
hypertension treatment. Rev Latino-Am Enfermagem..

[6] Coelho EB, Neto MM, Palhares R, Cardoso MCM, Geleilete TJM, Nobre F (2005). Relação entre a assiduidade às consultas ambulatoriais e
o controle da pressão arterial em pacientes hipertensos. Arq Bras Cardiol..

[7] Grezzana GB, Stein AT, Pellanda LC (2013). Adesão ao tratamento e controle da pressão arterial por
meio da monitoração ambulatorial de 24 horas. Arq Bras Cardiol..

[8] Carvalho ALM, Leopoldino RWD, Silva JEG, Cunha CP (2012). Adesão ao tratamento medicamentoso em usuários
cadastrados no Programa Hiperdia no município de Teresina (PI). Ciênc saúde coletiva..

[9] Demoner MS, Ramos ERP, Pereira ER (2012). Fatores associados à adesão ao tratamento
anti-hipertensivo em unidade básica de saúde. Acta Paul Enferm..

[10] Karakurt P, Kasikçi M (2012). Factors affecting medication adherence in patients with
hypertension. J Vasc Nurs..

[11] Martin MY, Kohler C, Kim Y, Kratt P, Schoenberger YM, Mark S (2010). Taking Less Than Prescribed: Medication Nonadherence and
Provider-Patient Relationships in Lower-Income, Rural Minority Adults With
Hypertension. J Clin Hypertens (Greenwich)..

[12] Monteiro CN, Farias RE, Alves MJM (2009). Perfil de hipertensos em populações urbana e rural no
estado de Minas Gerais. Rev APS..

[13] Instituto Brasileiro de Geografia e Estatística (IBGE) (2010). http://cidades.ibge.gov.br/xtras/temas.php?lang=&codmun=315690&idtema=1&search=minas-gerais|sacramento|censo-demografico-2010.

[14] Sociedade Brasileira de Cardiologia, Sociedade Brasileira de Hipertensão, Sociedade Brasileira de Nefrologia (2010). VI Diretrizes Brasileiras de Hipertensão
Arterial. Rev Bras Hipertens..

[15] Associação Brasileira de Empresas de Pesquisa - ABEP (2014). Critério de Classificação Econômica Brasil.

[16] Santa Helena ET, Nemes MI, Eluf-Neto J (2010). Evaluation of care provided for people with arterial
hypertension in family health strategy services. Saúde Soc..

[17] McDonald MV, Pezzin LE, Peng TR, Feldman PH (2009). Understanding the complexity of hypertensive african
american home care patients: challenges to intervention. Ethn Dis..

[18] Rolnick SJ, Pawloski PA, Hedblom BD, Asche SE, Bruzek RJ (2013). Patient characteristics associated with medication
adherence. Clin Med Res..

[19] Vitor AF, Monteiro FP, Morais HC, Vasconcelos JD, Lopes MV, Araújo TL (2011). Survey of the follow-therapeutic patients with
hypertension. Esc Anna Nery Rev Enferm..

[20] Andrade JP, Vilas-Boas F, Chagas H, Andrade M (2002). Epidemiological aspects of adherence to the treatment of
hypertension. Arq Bras Cardiol..

[21] Amado Guirado E, Pujol Ribera E, Pacheco Huergo V, Borras JM, ADIEHTA Group (2011). Knowledge and adherence to antihypertenive therapy in
primary care: results of a randomized trial. Gac Sanit..

[22] Mion D, Pierin AMG, Ignez EC, Marcondes M (1996). Non-pharmacological treatment: low level of awareness in
a developing country. Am J Hypertens..

[23] Rasella D, Harhay MO, Pamponet ML, Aquino R, Barreto ML (2014). Impact of primary health care on mortality from heart
and cerebrovascular diseases in Brazil: a Nationwide analysis of longitudinal
data. BMJ..

